# Neuronal injury and hepatotoxicity: astrocytes and stellate cells convergence and their role in tissue repair

**DOI:** 10.3389/fnins.2025.1733612

**Published:** 2026-02-03

**Authors:** Ana Catya Jimenez-Torres, Arturo Ortega, Mustapha Najimi

**Affiliations:** 1 Department of Pharmacology and Toxicology, Medical College of Georgia, Augusta University, Augusta, GA, United States; 2 Departamento de Toxicología, Centro de Investigación y de Estudios Avanzados del Instituto Politécnico Nacional (Cinvestav-IPN), Mexico City, Mexico; 3 Laboratory of Pediatric Hepatology and Cell Therapy, Institute of Experimental and Clinical Research (IREC), UCLouvain, Brussels, Belgium

**Keywords:** astrogliosis, brain-liver axis, hepatic stellate cells, liver repair, transcriptome, liver diseases

## Abstract

In the central nervous system, astrocytes are highly specialized non-neuronal cells that are key elements in maintaining neuronal microenvironment homeostasis. These cells provide structural and metabolic support to other brain cells and regulate ion concentrations and the local levels of neurotransmitters such as glutamate. Astrogliosis, characterized by morphological and functional abnormalities, has been implicated in various neuronal disorders. Similarly, hepatic stellate cells drive the initiation and the progression of liver fibrosis. After liver injury, hepatic stellate cells are activated through inflammatory mediators and differentiate into activated myofibroblasts. Hepatic stellate cells express several glial-related molecules, suggesting functional similarities between these two cell types, which paves the way for a better understanding of crucial targets for neuronal and liver repair. We present herein a compressive update of our current knowledge of the transcriptome of activated hepatic stellate cells during liver injury and contrast it with that of reactive astrocytes in neuronal diseases. Furthermore, we summarize the plausible involvement of long non-coding and microRNAs in the transcriptional regulation of specific genes during neuronal and liver injuries. Finally, we discuss possible common targets and novel strategies to diminish the activation of stellate cells and astrocytes as therapeutic strategies. In addition, we highlight new insights into the brain-liver axis.

## 1 Introduction

The close connection between the central nervous system (CNS) and the liver has been underlined recently. The so-called brain-liver axis points to a dynamic communication of their metabolic, hormonal, and immunological connections (Sun X. et al., 2024). Moreover, similarities in gene expression patterns between specific liver and brain cell types highlight not the parallelism of both organs in the maintenance of physiological functions in response to injury (Schachtrup et al., 2011). Reactive gliosis and activation of hepatic stellate cells (HSCs) contribute to the adaptative response to different stimuli, driving the development of various diseases in the liver and the CNS. Although the brain is not completely analogous to the liver environment, the presence of key transcripts in astrocytes and HSCs contribute to envision shared molecular mechanisms in response to injury in both cell types. Transcription factors are the most common way to control gene expression by decoding the DNA sequence (Ratti et al., 2020). It is well known that regulatory networks are dynamic across different cell types; the same transcription factor can regulate different genes. However, mapping and identifying highly specific expression programs in the transcriptome might provide a comprehensive perspective on the molecular mechanisms by which a particular transcription factor targets the same genes in different cell types, such as astrocytes and HSCs. The transcriptional machinery can be regulated by a large family of non-coding RNAs [long non-coding (lncRNAS) and micro RNAs] as regulators of imprinting, cell cycle, pluripotency, development, and the immune response (Ratti et al., 2020; Wilusz et al., 2009). Moreover, it has been demonstrated that crosstalk between microRNA and lncRNA regulates gene expression at the transcriptional level and is involved in pathological conditions.

This review aims to focus on the transcriptional profile of reactive astrocytes and activated HSCs. We discuss the atlas of both cells across neuronal and liver disease etiologies that may predict a specific signature through the so-called “brain-liver axis” to identify novel therapeutic targets for treating neuronal and hepatic diseases.

## 2 Physiological basis of brain-liver axis

**FIGURE 1:**
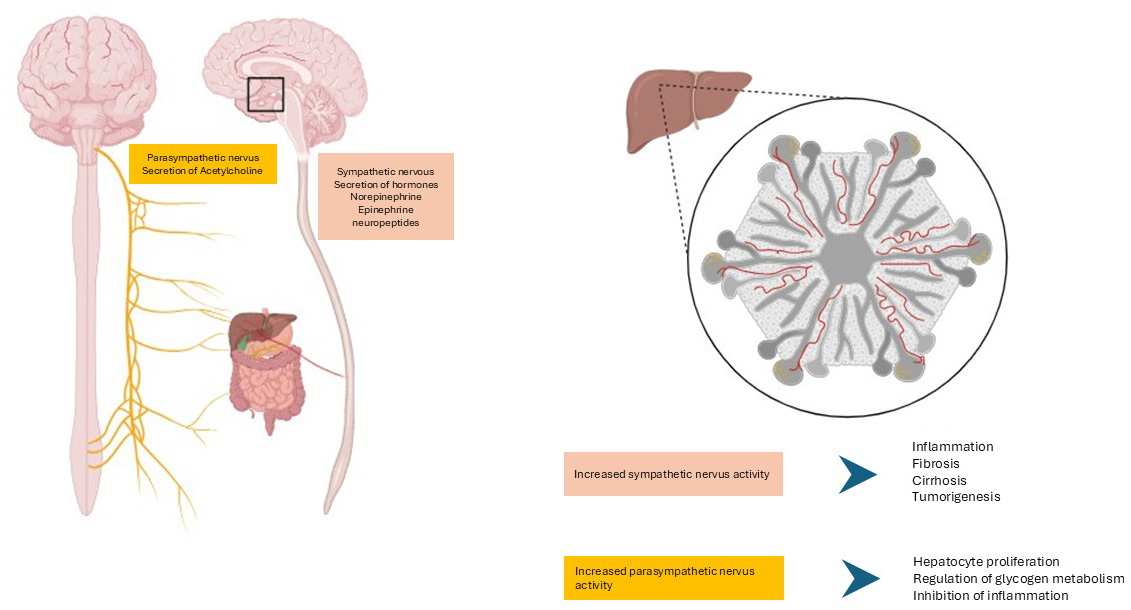
Anatomical and physiological basis of the brain- liver axis. Sympathetic and parasympathetic nerves modulate the autonomic hepatic regulation. Sympathetic nerves project on hepatocytes in the human liver parenchyma meanwhile the parasympathetic nerves terminate at Disse’s space near HSCs and sinusoidal endothelial cells. Figure created with BioRender.com.

Anatomically, the brain-liver axis is defined by nerve fiber connections between both organs. One of these networks are placed from the hypothalamus to the Disse’s space in the liver, sympathetic and parasympathetic nerves modulate the autonomic hepatic regulation (Figure 1; Luo et al., 2023; Ma et al., 2025; Uyama et al., 2004). Electrical stimulation of hypothalamic regions such as the ventromedial and lateral nuclei regulates glycogenolysis and gluconeogenesis in the liver (Veneziale et al., 1967). Since Shimazu et al. (1966) supported that the sympathetic pathway between the ventromedial hypothalamic nuclei and the liver controls the glycogen content in this organ. Stimulation of the ventromedial hypothalamic nuclei induces the activation of the glycogenolytic liver enzymes: glycogen phosphorylase and glucose-6- phosphatase resulted in reduced glycogen content in the liver and the increase of glucose in blood (Shimazu et al., 1966; Veneziale et al., 1967). Meanwhile, the chemical or electrical stimulation of the lateral hypothalamic nuclei which is connected to the liver through the vagus nerve, induces the activation of liver glycogen synthase, key enzyme controlling glycogen metabolism, indicating the crucial crosstalk in the hypothalamic parasympathetic nervous system as well (Ma et al., 2025; Uyama et al., 2004; Veneziale et al., 1967). In both cases, the direct neural effect on the hepatic energy homeostasis was determined under pancreatectomy and adrenalectomy, which do not prevent the effects on the activation of the liver enzymes (Shimazu et al., 1966; Veneziale et al., 1967).

The medulla oblongata is the other brainstem region associated with the vagus nerve in the brain- liver axis. The dorsal motor nucleus of the vagus nerve begins at the parasympathetic neurons within the medulla oblongata and projects to the liver through the hepatic branch via the hepatic artery (Ma et al., 2025). This parasympathetic innervation has been described in mammals. In contrast with the sympathetic nerves which synapse on hepatocytes in the liver parenchyma in many mammals except in mouse, rat and golden hamster, the parasympathetic nerves terminate at Disse’s space near hepatic stellate cells (HSCs) and sinusoidal endothelial cells (Miller et al., 2021). Thus, the signals in the brain-liver axis may occur by direct or indirect innervation. By direct innervation for example the imbalance in the secretion of hormones such as epinephrine and norepinephrine by the sympathetic nerve induces the release of the transforming grow factor β and the production of collagen in HSCs, the secretion of interleukin-6 by Kupffer cells via the α/β adrenergic and neuropeptide Y receptors promoting liver inflammation, the activation and proliferation of HSCs changing the liver microenvironment contributing to liver fibrosis, cirrhosis and tumorigenesis (Amir et al., 2020; Cawley et al., 2014; Luo et al., 2023; Miller et al., 2021). Meanwhile the secretion of acetylcholine by the terminals of the parasympathetic nerve diminishes hepatic inflammation and hepatocyte apoptosis contributing to hepatocyte and hepatic progenitor cell proliferation. The indirect metabolic regulation via signal transmission occurs because the sinusoidal innervation that communicates endothelial and Kupffer cells with hepatocytes and stellate cells (Miller et al., 2021). Cytokines and small polypeptides such as neurotropic factors, GDNF, BDNF, NRG4 and CNTF play a key role in the regulation of the hepatic nervous system.

As part of the indirect signaling in the brain- liver axis, the crosstalk between the activation of glial cells in the central nervous system and the activation of hepatic stellate cells in the regulation of hepatic lipid metabolism and matrix formation evidences that neuroendocrine system and neuroinflammation mediated by glial activation may be closely related to the initiation of fatty liver disease and liver fibrosis (Schachtrup et al., 2011; Wang et al., 2024).

*In vitro* studies have demonstrated neural features of HSCs, for example proliferation, collagen gene expression and pro-fibrotic state of HCSs can be regulated via sympathetic neurotransmitters such as norepinephrine and serotonin (Oben et al., 2003b,^2004^; Ruddell et al., 2006). Moreover, acetylcholine, neuropeptide Y and endocannabinoid modulate proliferation, cell death by necrosis and apoptosis in HSCs (Oben et al., 2003a, b; Siegmund et al., 2005). Specific neuroglial molecules are expressed in HSCs in both models, human and rat (Cassiman et al., 2001); although all the functions in this cell type are still not fully studied, it has been reported that neurotrophins such as NGF, BDNF, NT-3 and 4, TrK receptors, the glial fibrillary acid protein (GFAP), neural cell adhesion molecule (NCAM), p75*^NTR^*, nestin, synemin, hedgehog and nucleotide receptors mediated tissue remodeling by cell activation, differentiation, apoptosis and contraction of HSCs (Cassiman et al., 2001; Schachtrup et al., 2011).

Although glial cells, specifically, astrocytes and HSCs are not fully analogous models, the physiological, functional similarities and close communication via neuroendocrine signals suggest a novel approach by targeting both cell types into nervous system and liver restoration.

**FIGURE 2:**
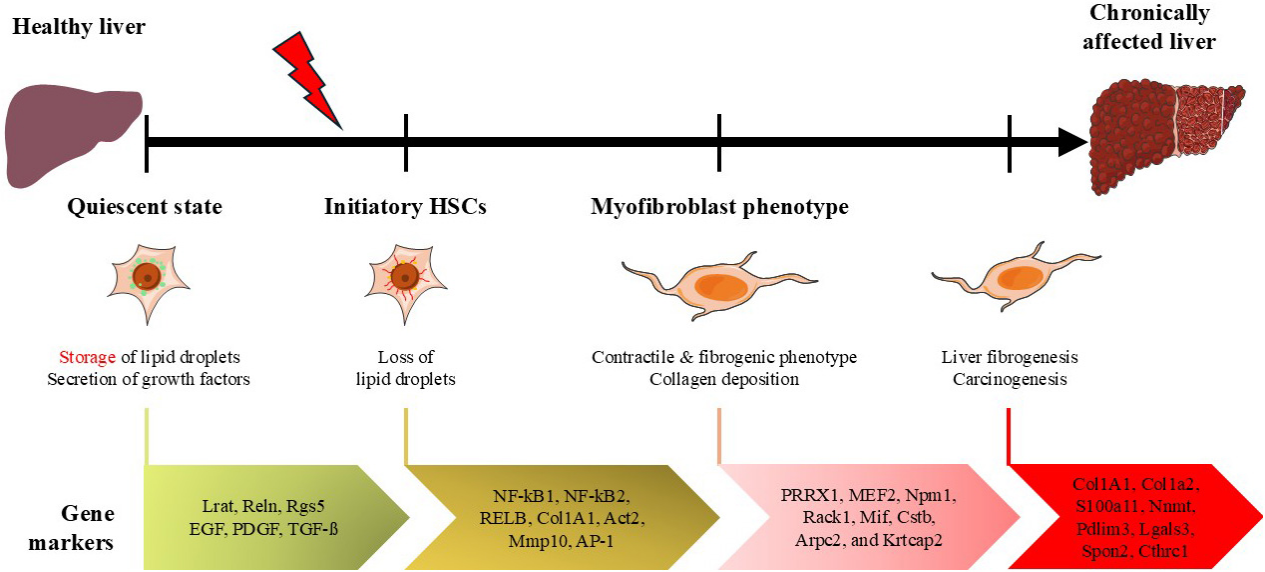
Overview of pseudotime trajectory of genes expressed in HCSs from quiescent state to myofibroblast-like phenotype. qHSCs maintain the liver microenvironment acting as reservoirs of lipids and by the release of growth factors. Under liver injury, the loss of lipid droplets occurs and if liver damage persists, HSCs acquire contractile and fibrogenic phenotype, increasing extracellular matrix components resulting in collagen deposition which promotes liver fibrogenesis and carcinogenesis. Figure adapted from Servier Medical Art (https://smart.servier.com), licensed under CC BY 4.0 (https://creativecommons.org/licenses/by/4.0/).

Astrocytes and HSCs are shaping the metabolic microenvironment in the brain and liver, respectively; both cell types might create conditions that control physiological conditions as well as immunological and metabolic reprogram and tumor promotion influencing either positively or negatively disease progression (Jaraíz-Rodríguez et al., 2023; Malone et al., 2024; Figure 2).

## 3 The atlas of hepatic stellate cells in liver injury

**FIGURE 3:**
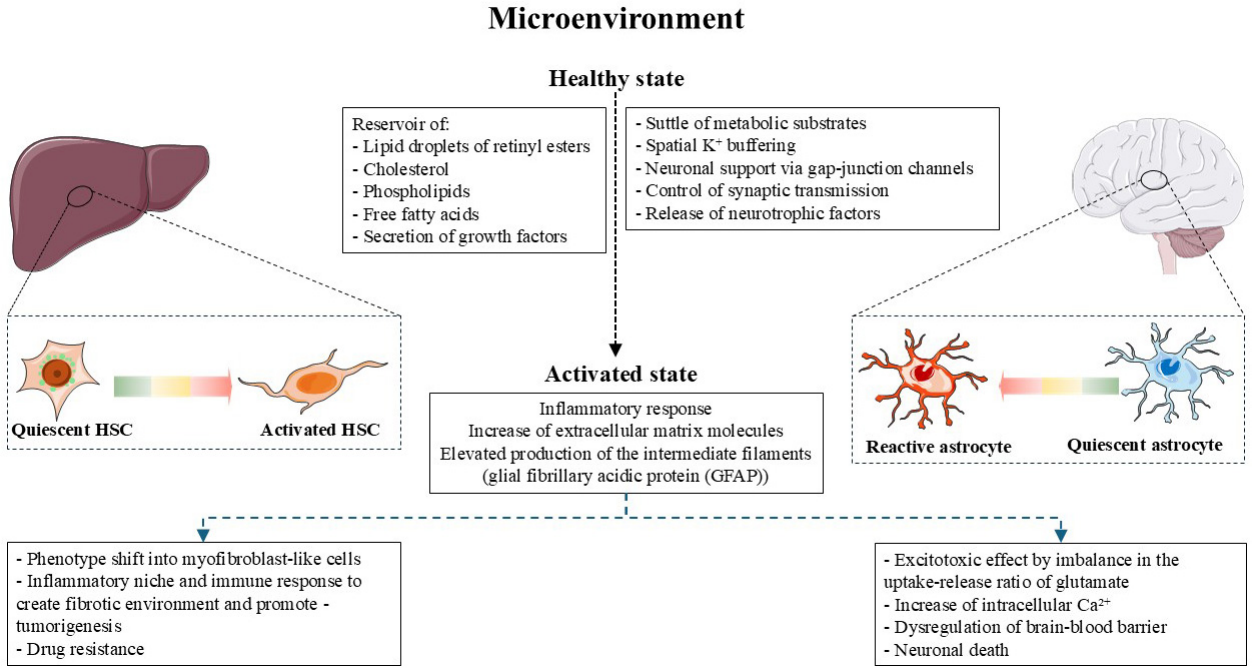
Microenvironment of HSCs and astrocytes. During physiological state HSCs act as reservoir of lipid droplets of retinyl esters, small amounts of cholesterol, phospholipids and free fatty acids; moreover, regulates the liver microenvironment by the secretion of growth factors. Meanwhile the astrocytes regulate the metabolic state of the central nervous system by shuttle glucose-derived lactate to neurons and regulating K^+^ buffering. Additionally, astrocytes control synaptic transmission by the clearance of neurotransmitters such as glutamate and the release of neurotrophic factors. During the activate/reactive state, both cell types induce inflammatory and immune response and increase molecules in the extracellular matrix resulting in pathological state in the liver and central nervous system. Figure adapted from Servier Medical Art (https://smart.servier.com), licensed under CC BY 4.0 (https://creativecommons.org/licenses/by/4.0/).

Although the HSCs comprise less than 8% of the normal liver cell population, these cells are involved in critical functions under physiological conditions and in response to liver injury (Mederacke et al., 2013; Wiering et al., 2023). The HCSs are located in the space of Disse in contact with sinusoidal endothelial cells and hepatocytes (Friedman, 2008). In healthy conditions, HCSs reside in a quiescent state, releasing growth factors to regulate hepatocyte regeneration and, in fact, represent the major reservoir of cytoplasmic lipid droplets of retinyl esters (vitamin A) and small amounts of cholesterol, phospholipids, and free fatty acids (Blaner et al., 2009). During liver injury, HSCs become activated and are the predominant source of fibrous extracellular matrix. Under such circumstances, they lose their lipid storage and differentiate into proliferative, fibrogenic, and contractile myofibroblasts, contributing to anarchic collagen deposition (Kamm and McCommis, 2022). The transcriptome of HSCs has been described in three different profiles: quiescent state (qHSCs), initiatory HSCs, and myofibroblast phenotype (Merens et al., 2025; Figure 3). Single-cell sequencing datasets have led to the construction of the HCSs atlas to understand the regulatory mechanisms of HCSs activation and its role in developing diverse liver injuries. Worldwide liver diseases contribute to approximately 2 million deaths. The incidence of non-alcoholic steatohepatitis (NASH) increased 94.49% in the last decade (Gu et al., 2025), which raised the probabilities of developing cirrhosis and liver cancer. By 2015 the global incidence of cirrhosis increased 13% with 23.4 per 100, 000 individuals (Gan et al., 2025). Meanwhile, liver cancer was ranked the six most frequent diagnosed cancer worldwide (7.8% of all cancers globally) by 2022, with 865, 269 new cases reported and 757,948 deaths indicating poor prognosis which representing a global health burden (Bray et al., 2024). In this section, we discuss the gene expression patterns of HCSs during various models of liver diseases such as non-alcoholic steatohepatitis, chronic hepatitis, fibrosis, cirrhosis, and cancer.

### 3.1 Non-alcoholic steatohepatitis

Non-alcoholic steatohepatitis is characterized by inflammation and hepatocellular injury; it plays a central role in the development of liver fibrosis, with a 40% incidence, making it the second leading cause of liver transplantation in the United States (Carter and Friedman, 2022; Noureddin et al., 2018). As the main fibrogenic cell type in the liver, the excess of the extracellular matrix *via* the activation of HSCs is a hallmark feature in the prognosis of patients with non-alcoholic fatty liver disease (NAFLD) (Hagström et al., 2017). Single-cell RNA-sequencing analysis (scRNASeq) of HSCs has identified a specific transcriptome signature in NASH patients (He et al., 2023). Sixty-one genes were enriched in the cell cluster of activated HSCs but not in the population of inactivated HSCs (He et al., 2023). Specifically, the expression of genes essential in cell-mediated immunity and anti-viral activity, such as interferon-induced transmembrane protein 1 (IFITM1) and macrophage migration inhibitory factor (MIF), is upregulated. Genes relevant for cell adhesion, cell proliferation, and cell-cell communication- such as collagen type 1 alpha 1 chain (*Col1A1*) and 2 chain (*Col1a2*), S100 calcium-binding protein A11 (*S100a11*), alpha-actinin-2-associated LIM protein (*Pdlim3*), galectin 3 (*Lgals3*), spondin 2 (*Spon2*), nicotinamide N-methyltransferase (*Nnmt*) and collagen triple helix repeat containing 1 (*Cthrc1*)- are augmented and correlated with the severity of fibrosis in NASH (Azevedo Foinquinos et al., 2020; Feng et al., 2021; He et al., 2023; Heinrichs et al., 2021; Hou et al., 2018; Qi et al., 2017; Shin et al., 2018; Sun J. et al., 2024; Teng et al., 2021; Wu C. et al., 2021; Younossi et al., 2009) (see Table 1). Moreover, epidermal growth factor (EGF), platelet derived growth factor (PDGF), and transforming growth factor beta (TGF-ß)-mediate HSCs activation (Ahmed et al., 2022; Bhushan et al., 2019; Wiering et al., 2023; Table 1). Tamoxifen-induced expression of PDGF subunit B (PDGF-B) in the liver of transgenic mice acts as a proliferative and profibrogenic stimulus, inducing transdifferentiation of HSCs; additionally, expression levels of MMP-2, MMP-9, and TIMP-1 are upregulated, with no significant changes in TGF-ß (Czochra et al., 2006; Table 1). This suggests a TGF-ß-independent mechanism in PDGF-B transgenic mice and highlights the importance of the specific markers across transition to activated HSCs in different models of liver disease. For example, the expression of PDGF can be up-regulated in response to Il-1α as well (Andrae et al., 2008). Interestingly, the gene encoding the expression of the PDGF receptor beta shows upregulation in inactivated HSCs in NASH but not in quiescent HSCs in healthy cell-clusters (He et al., 2023), suggesting a PDGF-dependent mechanism in the early stages of NASH followed by activation of HSCs and transition to fibrotic states.

**TABLE 1:** Transcriptome atlas of activated HSCs in liver diseases.

Liver disease	Gen	Regulation	Cellular function	References
Non-alcoholic steatohepatitis	*Ifitm1*	↑	Intercellular adhesion, cell growth, cell migration	He et al., 2023; Younossi et al., 2009
	*Mif*	↑	Inflammatory response	He et al., 2023; Heinrichs et al., 2021
	*Col1a1*	↑	Production of collagen type I	He et al., 2023; Qi et al., 2017
	*Col1a2*	↑	Production of collagen type I	He et al., 2023; Sun J. et al., 2024
	*S100a11*	↑	Lipid deposition	He et al., 2023; Teng et al., 2021
	*Pdlim3*	↑	Cytoskeletal structure	He et al., 2023; Wu C. et al., 2021
	*Lgals3*	↑	Inflammatory response, cell growth	Azevedo Foinquinos et al., 2020; He et al., 2023
	*Spon2*	↑	Immune response	He et al., 2023; Hou et al., 2018
	*Nnmt*	↑	Cell metabolism, epigenetic remodeling	He et al., 2023; Shin et al., 2018
	*Cthrc1*	↑	Cell contractility, cell migration	Feng et al., 2021; He et al., 2023
	*Efg*	↑	Lipogenesis	Bhushan et al., 2019; Wiering et al., 2023
	*Pdgf*	↑	Cell proliferation, fibrogenic response	Wiering et al., 2023
	*Tgf-ß*	↑	Inflammatory response, fibrogenesis	Ahmed et al., 2022; Wiering et al., 2023
	*Mmp-2*	↑	Extracellular matrix architecture	Czochra et al., 2006; Okazaki et al., 2014; Stefano et al., 2015
	*Mmp-9*	↑	Extracellular matrix architecture	Czochra et al., 2006; Okazaki et al., 2014; Stefano et al., 2015
	*Timp-1*	↑	Extracellular matrix architecture	Czochra et al., 2006
Liver fibrosis	*Fosl1*	↑	Extracellular matrix	Taha et al., 2023
	*Fosl2*	↑	Cytoskeletal rearrangements, immune response	Kim et al., 2024; Taha et al., 2023
	*Ap-1*	↑	Extracellular matrix remodeling	Kim et al., 2024; Rippe and Brenner, 2004; Taha et al., 2023
	*Nf-kß*	↑	Fibrogenesis, cell apoptosis, inflammatory response	Luedde and Schwabe, 2011; Shen et al., 2014; Wu Y. J. et al., 2021
	*Tnf*-α	↑	Extracellular matrix remodeling, immune response,	Yasmeen et al., 2023; Osawa et al., 2013
	*Wt1*	↑	Cell migration, fibrogenesis	Kendall et al., 2019; Merens et al., 2025
	*Pdgf*	↑	Cell migration	Gong et al., 2017; Merens et al., 2025
	*Mef2c*	↑	Fibrogenesis	Merens et al., 2025; Wang et al., 2004
HCC	*Lrat*	↓	Production of retinyl esters	Haaker et al., 2024; Wang H. et al., 2022
	*Reln*	↓	Extracellular matrix	Kobold et al., 2002; Wang H. et al., 2022
	*Rgs5*	↓	Cell contraction, cell migration, fibrogenesis	Bahrami et al., 2014; Wang H. et al., 2022
	*Acta2*	↑	Cell motility, cell contraction	Rockey et al., 2013; Wang H. et al., 2022
	*Ccn2*	↑	Fibrotic response	Pi et al., 2023; Rachfal and Brigstock, 2003; Wang H. et al., 2022
	*Mmp10*	↑	Extracellular matrix remodeling, fibrotic response	Han, 2006; Knittel et al., 2000; Wang H. et al., 2022
	*Npm1*	↑	Cell proliferation/ inhibition of apoptosis	Ding et al., 2023; Wang H. et al., 2022
	*Rack1*	↑	Cell proliferation, cell differentiation, cell migration, fibrogenesis	Bourd-Boittin et al., 2008; Liu et al., 2015; Wang H. et al., 2022
	*Mif*	↑	Immune response, cell migration	Qin et al., 2021; Wang H. et al., 2022
	*Cstb*	↑	Cell proliferation, fibrotic response	Moles et al., 2009; Wang H. et al., 2022
	*Arpc2*	↑	Cell migration, cellular structure	Huang et al., 2021; Wang H. et al., 2022
	*Krtcap2*	↑	Immune response	Sun et al., 2023; Wang H. et al., 2022

↑Upregulation in activated HSCs. ↓Downregulation in activated HSCs.

### 3.2 Liver fibrosis

The transcriptional signature of qHSCs differentiation into myofibroblasts shows high activity of the transcription factors FOS like 1 (FOSL1) and FOSL2, members of the activator protein-1 complex (AP-1) (Kim et al., 2024; Rippe and Brenner, 2004; Taha et al., 2023). Overexpression of *Fosl1* promotes spontaneous liver fibrosis in mouse models and is associated with the progression of liver tumors and worse prognosis in patients with hepatocellular carcinoma (HCC) (Taha et al., 2023). Members of the Nuclear Factor-kß- (NF-kß) family, such as NF-kB1, NF-kB2, and RELB, are highly active at early stages of HSCs activation. NF-kß plays a key role in hepatic injury, particularly in the transition of fibrosis to HCC (Luedde and Schwabe, 2011; Shen et al., 2014; Wu Y. J. et al., 2021). The mammalian NF-kß family dimers comprise the interaction of five subunits: p50, p52, cRel, p65 (also known as RelA) and RelB, encoded by NF-κB1, NF-κB2, REL, RELA, and RELB, respectively (Ghosh and Hayden, 2008; Hoffmann et al., 2003). NF-kß activation may occur *via* canonical and non-canonical pathways. The canonical NF-kß pathway is activated in response to inflammatory stimuli and is related to the activation of HSCs (Elsharkawy et al., 2010). Various drugs including *Sofosbuvir* and *Velpatasvir* display antifibrotic effects in carbon tetrachloride (CCl_4_)-induced fibrosis rat model; these effects are not dependent on their antiviral activity but are mediated through the suppression of HSCs via regulation of TNF-α levels and its downstream NF-κB pathway (Yasmeen et al., 2023). The activity patterns of the transcription factor WT1, the paired related homeobox protein 1 (PRRX1), and the transcription factor myocyte enhancer factor 2 (MEF2) demonstrate high activation in myofibroblasts in both mouse and human cells (Kendall et al., 2019; Merens et al., 2025; Wang et al., 2004). For example, Prrx1 is involved in PDGF-dependent HSCs migration *via* modulation of metalloproteinases MMP2 and MMP9 expression. Moreover, administration of an adenoviral-mediated *Prrx1* shRNA attenuates liver fibrosis induced by thioacetamide in rats (Gong et al., 2017). Similarly, *Mef2* interference RNA significantly inhibits the expression of smooth muscle- α (α-SMA), COL1A1, and proliferating cell nuclear antigen, all markers in liver fibrogenesis. Collectively, these findings suggest that the upregulation of these genes is conserved between humans and mice and that they play pivotal roles in HSCs activation across different liver injury models (Table 1).

### 3.3 Hepatocellular carcinoma

In the last two decades, the HCC mortality rate has increased globally (Singal et al., 2023). In both primary and metastatic liver cancers, HSCs are the main source of activated myofibroblast-like cells (Cogliati et al., 2023). The scRNASeq data from fibrotic mouse liver (GSE1326620), human cell populations of patients with NAFLD (GSE49541), and patients with liver fibrosis during HBV infection (GSE89632, GSE84044), were retrieved from the Gene Expression Omnibus (GEO) dataset. The *in-silico* reconstruction of a single-lineage pseudo-time trajectory of HSCs activation identified three pseudotime-dependent differentiation stages. After the quiescent cell type (stage 1), HSCs display two diverse stages (stages 2 and 3) during *in vitro* transdifferentiation process (Wang H. et al., 2022). The downregulation of qHSCs markers such *Lrat*, *Reln*, and *Rgs5*, along with high expression levels of *Acta2*, *Ccn2*, and *Mmp10*- markers of activated HSCs- are characteristic of the early stages of HSCs-to-myofibroblast transition (state 2). For example, LRAT encodes the main enzyme in retinyl esters production in qHSCs, specifically lecithin: retinol acyltransferase (LRAT). The loss of retinyl esters is characteristic of activated HSCs; this disruption in retinyl ester metabolism is mediated by reduced levels of *Lrat*, loss of LRAT activity, and enhanced breakdown of retinyl esters (Haaker et al., 2024; Wang H. et al., 2022). Reelin, an extracellular matrix protein, has been studied *in silico* and *in vivo*, demonstrating lower levels of reelin in activated HCSs compared to qHSCs. Meanwhile, reelin expression is not detectable in rat liver myofibroblasts, indicating reelin as a key protein for distinguishing the transdifferentiation states of HSCs (Kobold et al., 2002; Wang H. et al., 2022). The regulator of G-protein signaling-5 (RGS5) is encoded by *Rgs5*. RGS5 controls contraction, migration, and fibrosis in HSCs by regulating G-protein coupled receptor (GPCR)-mediated signaling, via endothelin-1 (ET-1) and angiotensin II (AngII) (Bahrami et al., 2014; Wang H. et al., 2022). Interestingly, the levels of activated HSC markers show an increased pattern at the early stages of differentiation (state 2). Smooth muscle α actin (*Act2*) is undetectable in qHSCs isolated from normal livers but is abundant in activated HSCs, where it reduces cell motility and contraction (Rockey et al., 2013; Wang H. et al., 2022). The cellular communication network factor 2 (*Ccn2*)/connective tissue growth factor (*Ctgf*) (CCN2/CTGF), an extracellular signaling modulator, and the Slit2 ligand synergistically mediate HSC activation and fibrotic response in CCl_4_-induced liver injury by activating phosphatidylinositol 3-kinase (PI3K) and AKT signaling pathways. *In vitro* studies suggest that the production of CTGF/CCN2 is primarily regulated by TGF-β (Pi et al., 2023; Rachfal and Brigstock, 2003; Wang H. et al., 2022). The matrix metalloproteinase 10 (*Mmp10*) is also involved in fibrosis progression in the liver. The increase of MMP10 expression can be detected after acute liver damage, for example, following a single dose of CCl_4_; the secretion of MMPs degrades the normal extracellular matrix, leading to the activation of HSCs (Han, 2006; Knittel et al., 2000; Wang H. et al., 2022; Table 1).

Genes involved in liver carcinogenesis are upregulated in stage 3, when HSCs are predominantly differentiated into myofibroblasts (Wang H. et al., 2022). Interestingly, Npm1, Rack1, Mif, Cstb, Arpc2, and Krtcap2, crucial in cell proliferation, differentiation and migration, fibrogenesis, immune response, cell-cell junction, and HCC metastasis (Bourd-Boittin et al., 2008; Ding et al., 2023; Huang et al., 2021; Liu et al., 2015; Moles et al., 2009; Qin et al., 2021; Sun et al., 2023; Wang H. et al., 2022), are expressed at much higher levels in activated HSCs derived from cancer-associated fibroblasts than in diet biliary fibrosis or mice biliary fibrosis model (Wang H. et al., 2022), highlighting these specific genes as possible early indicators of the liver tumor microenvironment (Table 1). Moreover, genes associated with the regulation of signal transduction by p53 class mediators- essential for DNA damage response, intrinsic apoptotic signal, and regulation of protein ubiquitination- constitute more than 30% of the genes overexpressed in clusters of activated HSCs (Wang H. et al., 2022).

## 4 The atlas of astrocytes in nervous system diseases

The highly specialized glial cells, astrocytes, are involved in various functions in the brain, including the maintenance of the blood-brain barrier integrity (Abbott et al., 2006; Heithoff et al., 2021), ion homeostasis in the neuronal microenvironment, regulation of neurotransmitters such as glutamate and GABA, neuronal excitability, and plasticity through metabolic coupling with neurons. Because of their essential role in providing physical, energetic, and nutritional support to neurons and surrounding cells in the brain, astrocytes are key targets in the etiology of neurological disorders such as Alzheimer’s disease (AD), Parkinson’s disease (PD), Huntington’s disease (HD), epilepsy, depression, schizophrenia, and hepatic encephalopathy (HE) (Sepehrinezhad et al., 2023).

In the normal brain, astrocytes display transcriptomic heterogeneity across different brain regions (Boisvert et al., 2018; Chai et al., 2017; Serrano-Pozo et al., 2024). For example, studies in mice have described significant astrocyte diversity between the hippocampal and striatal circuits. Populations of striatal astrocytes exhibit “*per se*” enriched genes related to the cell cycle, cell proliferation, or chromosome structure, such as *Fam64a*, *Fzd5*, *Esco2*, *Sgo1*, *Kif18b*, *Ttk*, *Cdc20*, *Cdk1*. Meanwhile, genes involved in extracellular structure organization, synapse organization, biogenesis, neuronal differentiation and adult neurogenesis- such as *Dsp*, *Zic2*, *Serpinf1 and 2*, *Paupar, Zic1*, *Hopx*, *Angpt1*, *Cald1*, *Cav1*, *Gpnmb*- are enriched in hippocampal astrocytes (Chai et al., 2017). However, specific genes that encode proteins involved in the homeostasis of synaptic transmission- such as the glutamate aspartate transporter (*Slc1a3*, GLAST), glutamate transporter (*Slc1a2*, Glt-1), glutamine synthetase (*Glul*, GS), and the γ-aminobutyric acid transporter (*Slc6a11*, GAT3)- maintain similar expression levels in cortical, hypothalamic and cerebellar astrocyte populations (Boisvert et al., 2018). The latest report this year indicates that more than 57 million people live with neurodegenerative disease (Imam et al., 2025), resulting in cognitive and physical disability, low quality life and productivity of patients representing a high economic burden. In 2023, only in United States, approximately 6.7 million individuals were diagnosed with AD (Giri et al., 2024). Based on recent worldwide projections, an estimated 25.2 million people may be living with PD in 2050 (Su et al., 2025). Here, we describe the different transcriptomic profiles observed in various neurocognitive disorders, highlighting the genes involved in astrocyte reactivity.

### 4.1 Alzheimer’s disease

The etiology of AD remains unclear, but hallmark lesions in AD are characterized by abnormal folding and aggregation of amyloid-ß (Aß) and Tau proteins (Breijyeh and Karaman, 2020; Cummings et al., 1998). Astrocytes are involved in the pathophysiology of AD (Breijyeh and Karaman, 2020; Cai et al., 2017; Verkhratsky et al., 2010). Early studies on AD have shown an abundant population of glial cells at the neuritic plaques. It is now well established that reactive astrogliosis at the late stages of AD is a pathological modification observed in both the human brain and tissues isolated from AD animal models (Verkhratsky et al., 2010). Sequencing of single-nucleus RNA from astrocytes in brain regions representing the hierarchical spreading of pTau neurofibrillary tangles (NFB) along neural networks (i.e., using the Braak NFT staging system: entorhinal region, inferior temporal gyrus, dorsolateral prefrontal cortex, secondary and primary visual cortex), reveals transcriptomic changes in astrocytes throughout the temporal progression of AD in human brains (Serrano-Pozo et al., 2024). The expression of relevant genes in cell energy metabolism (Aldh2, Ckb, Pfkb), lipid metabolism (Apoe, Lrp4), cell-cell communication and mitochondrial function (Gjal1), and intracellular transport (Atp2b4, Slc27a1, Slc38a2, Slc39a11, Slc39a12, Trak1) is low at early and intermediate stages, peaks at the late stages of AD, and then decreases expression without returning to baseline at the end of the stage characterized by moderate NPs (Bhalla et al., 2023; Lv et al., 2015; Ren et al., 2020; Serrano-Pozo et al., 2024; Sun et al., 2016; Wilson et al., 1997; Zheng et al., 2024; Table 2). However, specific genes are upregulated in astrocytes at late stages of AD. These include genes that encode heat shock proteins (HSP90AA1, HSP90AB1, HSPA1A, HSPA1B, HSPA4, HSPA4L, HSPA8, HSPA9, HSPB1, HSPD1, HSPH1), antioxidant response (NFE2L2, PRDX1, SOD1, SOD2), inflammatory response (IL17RB, NFAT5), translation factors (EEF1A1, EIF1, EIF2S2), cytoskeleton and extracellular matrix proteins (CLIP2, MAP2, VIM, MMP16, PLOD2 and 3, SERPINH1, ST6GALNAC6), and glutamate metabolism (GS, Glt-1) (Escartin et al., 2021; Geisert et al., 1990; Nakano-Kobayashi et al., 2023; Serrano-Pozo et al., 2024; Szeliga, 2020; Table 2). These findings demonstrate that astrocytes exhibit a strong response to various types of stress in AD.

**TABLE 2:** Transcriptome atlas of reactive astrocytes in neurocognitive disorders.

Neurocognitive disease	Gen	Regulation	Cellular function	References
Alzheimer’s disease	*Aldh2*	↓	Energy metabolism GABA synthesis	Bhalla et al., 2023; Serrano-Pozo et al., 2024
	*Ckb*	↓	Energy metabolism	Serrano-Pozo et al., 2024; Wilson et al., 1997; Zheng et al., 2024
	*Pfkb*	↓	Energy metabolism	Lv et al., 2015; Serrano-Pozo et al., 2024
	*Apoe*	↓	Lipid metabolism	Serrano-Pozo et al., 2024
	*Lrp4*	↓	Neuromuscular junction cell metabolism Glutamatergic neurotransmission	Serrano-Pozo et al., 2024; Sun et al., 2016
	*Gja1*	↓	Cell-cell communication Mitochondrial function	Ren et al., 2020; Serrano-Pozo et al., 2024
	*Atp2b4, Slc27a1, Slc38a2, Slc39a11, Slc39a12*, *Trak1*	↓	Intracellular transport	Serrano-Pozo et al., 2024
	*Hsp90aa1, Hsp90ab1, Hspa1a, Hspa1b, Hspa4, Hspa4l, Hspa4l, Hspa8, Hspa9, Hspb1, Hspd1, Hsph1*	↑	Protein folding	Serrano-Pozo et al., 2024
	*Nfe2l2*	↑	Antioxidant response	Nakano-Kobayashi et al., 2023; Serrano-Pozo et al., 2024
	*Prdx1*	↑	Antioxidant response, apoptosis	Serrano-Pozo et al., 2024; Szeliga, 2020
	*Sod1*	↑	Antioxidant response,	Serrano-Pozo et al., 2024
	*Sod2*	↑	Antioxidant response	Serrano-Pozo et al., 2024
	*Il17rb*	↑	Inflammatory response, glutamatergic neurotransmission	Serrano-Pozo et al., 2024; Kostic et al., 2017
	*Nfat5*	↑	Inflammatory response, cell volume regulation	Serrano-Pozo et al., 2024; He et al., 2024
	*Eef1a1, Eif1, Eif2s2*	↑	Neuroinflammatory response	Serrano-Pozo et al., 2024; Aisha et al., 2023
	*Clip2*	↑	Cell cytoskeleton	Serrano-Pozo et al., 2024
	*Map2*	↑	Cell cytoskeleton	Geisert et al., 1990; Serrano-Pozo et al., 2024
	*Vim*	↑	Cell cytoskeleton	Escartin et al., 2021; Serrano-Pozo et al., 2024
	*Mmp16*	↑	Cell cytoskeleton	Serrano-Pozo et al., 2024
	*Plod2/3*	↑	Cell cytoskeleton	Serrano-Pozo et al., 2024
	*Serpinh1*	↑	Cell cytoskeleton	Serrano-Pozo et al., 2024
	*St6galnac6*	↑	Cell cytoskeleton	Serrano-Pozo et al., 2024
	*Glul*	↑	Glutamate metabolism	Serrano-Pozo et al., 2024
	*Slc1a2*	↑	Glutamatergic neurotransmission, glutamate metabolism	Serrano-Pozo et al., 2024
Parkinson’s disease	*Park7*	↑	Oxidative response, inflammatory response	Helgueta et al., 2024; Kam et al., 2020
	*Slc1a2*	↓	Glutamatergic neurotransmission, glutamate metabolism	Kam et al., 2020; Kim et al., 2016
	*Snca*	↑ Missense mutation	Cell cytoskeleton, α-synuclein aggregation	An et al., 2021; Gu et al., 2010; Konno et al., 2016; Ozoran and Srinivasan, 2023
	*Gfap*	↑	Cell structure, cell-cell communication, cell migration	Gong et al., 2025
	*Serpina3*	↑	Cell apoptosis, extracellular matrix remodeling, inflammatory response	Akbor et al., 2021; Gong et al., 2025; Zattoni et al., 2022
	*Aqp4*	↑↓	Cell water homeostasis, regulation of small uncharged solutes	Binder et al., 2012; Gong et al., 2025; Ren et al., 2013; Simon and Iliff, 2016
	*Chi3l1*	↑	Immune response, cell proliferation	Chen A. et al., 2021; Gong et al., 2025; Ku et al., 2011; Yeo et al., 2019
Hepatic encephalopathy	*Gfap*	↑	Cell structure, inflammatory response	Elsherbini et al., 2022
	*Aqp4*	↑	Cell water homeostasis	Elsherbini et al., 2022
	*Tnf*α	↑	Inflammatory response	Balzano et al., 2020; Elsherbini et al., 2022
	*Rp110*	↑	Iron transport	Kim et al., 2022
	*Mc4r*	↑	Cell energy	Kim et al., 2022
	*Lrp8*	↓	Synaptic plasticity	Kim et al., 2022; Passarella et al., 2022
	*Mapk8*	↓	Cell apoptosis	Kim et al., 2022
	*Bdnf*	↓	Synaptic plasticity	Kim et al., 2022

↑Upregulation in astrogliosis. ↓Downregulation in astrogliosis.

### 4.2 Parkinson’s disease

A pathological feature of PD is the degeneration and loss of dopaminergic neurons in the substantia nigra pars compacta (Booth et al., 2017). However, it has been demonstrated that astrocyte dysfunction may also be involved in the pathogenesis of PD (Booth et al., 2017; Kam et al., 2020). Specific genes have been shown to have higher expression in astrocytes from postmortem human brain tissue, such as *Park7*, which is involved in the response to oxidative stress. Deletion or mutations in *Park7* increase sensitivity to oxidative stress and proinflammatory responses, disrupt extracellular matrix interaction, increase microglial activation, and impair glutamate uptake via EAAT2 (*Slc1a2*) (De Miranda et al., 2018; Helgueta et al., 2024; Kam et al., 2020; Kim et al., 2016; Table 2). The gene that encodes to α-synuclein protein, *Snca*, is considered a key player in PD. Mutations in the *Snca* gene may lead to early onset of PD. The neuropathological characteristics observed in postmortem brain samples from patients with *Snca* duplication mutations reveal histopathological damage in the locus ceruleus, the dorsal motor nucleus of the vagus, and the basal nucleus of Meynert, with aggregation of α-synuclein in the form of Lewy bodies and Lewy neurites, as well as a loss of dopaminergic neurons in the substantia nigra, amygdala and hippocampus, which are hallmarks of PD (Konno et al., 2016). Importantly, the aggregation of α-synuclein through phagocytic uptake, secretion of exosomes, transfer via tunneling nanotubules, and *de novo* aggregation has been reported in astrocytes (Ozoran and Srinivasan, 2023). This leads to chronic inflammation, astrocyte reactivity, and a reduction in their functional activity related to glutamate uptake, which correlates with the exacerbation of PD pathology (An et al., 2021; Gu et al., 2010). Recently, scRNA-seq profiles within the substantia nigra of PD samples identified that *Gfap*, *Serpina3*, *Aqp4*, and *Chi3l1* genes were upregulated in astrocyte populations with PD (Gong et al., 2025; Table 2). The water channel aquaporin-4 (AQP4) encoded by the *Aqp4* gene (Jung et al., 1994), is the most abundant aquaporin and is highly expressed in astrocytes endfoot, facilitating the bidirectional flow of water, cerebrospinal fluid, and small uncharged solutes from the brain parenchyma, making it a key player in water homeostasis in the CNS (Jung et al., 1994; Salman et al., 2022; Simon and Iliff, 2016). Intriguingly, changes or loss of AQP4 in perivascular locations have been reported not only in PD but also in AD, traumatic brain injury and epilepsy (Binder et al., 2012; Ren et al., 2013; Simon and Iliff, 2016). Although oligodendrocytes are considered the primary source of SERPINA3N protein, dysregulation of the *Serpina3* gene in astrocytes has been identified as a gene signature in AD and schizophrenia (Akbor et al., 2021; Zattoni et al., 2022). The role of SERPINA3 in neurological diseases is still not fully understood. It displays a cell-specific molecular mechanism but modulates blood-brain barrier integrity and neuronal cell death, and it is involved in the inflammatory response (Zhu et al., 2024). Interestingly, the glycoprotein Chitinase-3-like 1 protein (CHI3L1) is differentially expressed in the pseudotime trajectory in PD. This pattern is associated with astrocyte activation (Gong et al., 2025). CHI3L1 has been identified as a biomarker for the progression of neurocognitive disorders such as PD and multiple sclerosis (Gong et al., 2025; Song et al., 2024; Talaat et al., 2023; Yeo et al., 2019). It is secreted in response to immune activation, mainly by activated astrocytes in the CNS and its high expression is also related to glioma invasion and patient survival prognosis (Chen A. et al., 2021; Ku et al., 2011). In PD, CHI3L1 is highly expressed in astrocyte populations in the middle of the differentiation trajectory toward reactive astrocytes (Gong et al., 2025). The specific molecular mechanism of CHI3L1 in PD is not yet fully understood; however, it has been demonstrated that the intrinsic pathway of CHI3L1 in glioma involves the PI3K/AKT/mTOR pathway (Chen A. et al., 2021). CHI3L1 binds to the Receptor for Advanced Glycation End products (RAGE), activating the downstream ERK1/2-MAPK pathway, which is associated with cancer cell proliferation (Yeo et al., 2019). It has been suggested that CHI3L1 promotes the activation of the NF-κB pathway, leading to an inflammatory response and PD progression (Gong et al., 2025).

### 4.3 Hepatic encephalopathy

Hepatic encephalopathy (HE) is a brain dysfunction caused by acute or chronic liver insufficiency and/or portal-systemic shunting (Lu, 2023; Rose et al., 2020). HE is characterized by a spectrum of neurological and/or psychiatric abnormalities that may include acute changes in mental state, cognitive disturbances, motor impairment, sleep abnormalities, and, in severe cases, dementia, or a comatose state (Vilstrup et al., 2014). The pathophysiology of HE has not been fully elucidated; however, the elevation of toxins such as ammonia in the blood following liver disease- followed by the accumulation of neurotoxins (e.g., ammonia, manganese, inflammatory cytokines and glutamate) and metabolic impairment- contribute to the pathogenesis of HE (Lu, 2023). Interestingly, glial cells are a key target in HE. Early changes observed in experimental models of HE demonstrated cytoplasmic hypertrophy in astrocytes. Additionally, Alzheimer type II astrocyte change has been reported as a distinctive histopathological feature during the later phases of HE in human brain tissue (Norenberg, 1987).

Significative changes in astrocytic genes such as *Gfap*, *Aqp4*, *Tnf*α, and Kir 4.1 have been reported in the cerebral cortex in a HE rat model induced by thioacetamide (Elsherbini et al., 2022; Table 2). Similar to other neurocognitive diseases, the expression of *Gfap*, *Aqp4* and *Tnf*α genes increased in the brain tissue in HE, leading to gliosis, astrocyte swelling with enlarged nuclei, neuropil vacuolation, nuclear pyknosis in neurons, and an increase in brain water content (Elsherbini et al., 2022). Hyperammonemia, a hallmark in HE, increases the levels of *TNF*α through the activation and nuclear translocation of NF-κB in microglia, astrocytes and Purkinje neurons in both postmortem and rat cerebellar tissues (Balzano et al., 2020). This is correlated with an increase in GABAergic neurotransmission mediated by the activation of the TNFR1 receptor (Balzano et al., 2020; Cabrera-Pastor et al., 2018). The administration of cGMP reduces glial activation, neuroinflammation, and normalizes extracellular glutamate and GABA levels in the cerebellum, leading to the restoration of motor coordination in hyperammonemia and HE (Cabrera-Pastor et al., 2018). The cortical transcriptome profile in a mouse model of HE induced by bile duct ligation demonstrated an increase in genes that code for proteins related to iron transport (*Rp110*), energy expenditure, and insulin sensitivity (*Mc4r*). Meanwhile, proteins such as the low-density lipoprotein receptor-related protein 8 (*Lrp8*), MAPK8 mitogen-activated protein kinase 8 (*Mapk8*), brain-derived neurotrophic factor (*Bdnf*) are significantly decreased in HE (Kim et al., 2022; Table 2). Importantly, these protein expression patterns are closely related to AD and PD pathology. For example, LRP8, as a receptor of apolipoprotein E (ApoE) and Reelin, may initiate signal pathways crucial for synaptic plasticity through the tyrosine phosphorylation of the adaptor protein Dab1/2, followed by the activation of PI3K, ERK1/2, Src-family kinases and protein kinase B/Akt signaling cascades (Passarella et al., 2022). Moreover, ApoE, specifically the isoform ApoE4, is considered a genetic risk factor for sporadic AD. APOE4 induces a proinflammatory response by regulating Transgelin 3 expression and, ultimately, NF-kB activation in human astrocytes (Arnaud et al., 2022).

## 5 Non-protein-coding genes involved in HSCs and astroglial activation

The long noncoding RNAs (lncRNAs) and microRNAs (miRNAs) are two families of non-protein-coding genes. The interplay between miRNA and lncRNA is critical in gene expression regulation. This crosstalk regulates fundamental cellular events, including cell proliferation, differentiation, apoptosis, and immune response (Pathania et al., 2024). The evidence indicates that both miRNA and lncRNA are involved in liver diseases and neurocognitive disorders (Ghafouri-Fard et al., 2021; Jiang et al., 2024; Li et al., 2023; Qi et al., 2017; Wu Z. et al., 2021).

### 5.1 microRNAs and lncRNAs implicated in HSCs activation

Critical cell signaling pathways involved in the activation of HSCs and the progression of liver fibrosis- such as Wnt/ß-catenin, NF-κB, TGF-ß/Smad, Hedgehog, and Notch- are modulated by lncRNAs and miRNAs (Li et al., 2023; Wu Z. et al., 2021). As described in Section “2 Physiological basis of brain-liver axis, after liver injury,” qHSCs differentiate into activated HSCs. In this phase, it has been demonstrated that the microRNAs- namely miR-31, miR-17-5p, miR-19, miR-27b, miR-503, miR-103-3p, miR-130a/b, and miR-942- are highly expressed in liver fibrosis tissue and *in vitro* models of liver injury (Chen et al., 2020; Hu et al., 2015; Lu et al., 2015; Tao et al., 2020; Xie X. et al., 2021; Yu et al., 2015; Zhang et al., 2016; Zhu et al., 2018). These miRNAs promote the phenotypic differentiation of qHSCs into activated HSCs via TGF-ß, PI3K/AKT, and PPAR-_γ_ pathways (Li et al., 2023; Table 3). Interestingly, several studies have reported the downregulation of specific microRNAs in various fibrotic murine models, and inducing the expression of these microRNAs may lead to the suppression of HSC activation. miR-98, miR-30, miR-130a-3p, miR-146a-5p, miR-200a, miR-489-3p, miR-708, sja-miR-71a inhibit HSC activation via TGF-ß, PI3K/AKT, Wnt/ß-catenin, PPAR-_γ_, JAG1/Notch3, and Hedgehog signaling pathways (Du et al., 2015; Li L. et al., 2020; Li et al., 2021; Liu et al., 2021; Tu et al., 2015; Wang H. et al., 2020; Wang Q. et al., 2020; Yang et al., 2020; Table 3). After phenotypic differentiation, activated HSCs highly express α-SMA, collagen alpha 1 (COL1A), and GFAP, modifying the architecture of the extracellular matrix (Cequera, 2014; Zhao et al., 2024). During this phase, sja-miR-1, miR-140-3p, miR-195-3p up-regulate the expression of α-SMA and accumulation of extracellular matrix through the regulation of Wnt/ß-catenin and PI3K/AKT signaling (Li et al., 2023; Wang Y. et al., 2020; Wang A. et al., 2022; Wu et al., 2019). On the other hand, the role of LncRNAs has been reported in the development of liver cirrhosis and HCC progression by activating HSCs (Wu Z. et al., 2021). LncRNA-ATB competitively binds to the common miRNA responsive element of miR-425-5p with TGF-ß type II receptor (TGFBR2) and SMAD2, leading to activated HSCs and increased Col1A1 and α-SMA production (Fu et al., 2016). The upregulation of the lncRNA HOXA transcript at the distal tip (HOTTIP) has been described in human liver samples with liver fibrosis and cirrhosis as well as in liver tissue and HSC of CCl_4_ -treated mouse. HOTTIP negatively regulates miR-148a in a sequence-specific manner (Li Z. et al., 2018). miR-148a is involved in hepatocytic differentiation of progenitor cells (Jung et al., 2016). The downregulation of miR-148a-3p through direct interaction with HOTTIP (Han L. et al., 2020) leads to high levels of mRNA and protein expression of TGFBR1, TGFBR2, Smad2 and Smad3- regulators of HSC activation via the TGF-ß/SMAD pathways (Li Z. et al., 2018). The lncRNA small nucleolar RNA host gene 7 (SNHG7) and lncRNA plasmacytoma variant translocation 1 (PVT1) are considered potential oncogenes in HCC (Cui et al., 2017; Xie et al., 2020; Yang et al., 2019). Similar to HOTTIP, SNHG7 functions as a competing endogenous RNA (ceRNA). It has been reported that SNHG7 interacts with various miRNAs, including miR-9-5p, miR-29b, miR-122-5p, miR-216b, and miR-425. For example, SNHG7 binds to miR-29b and inhibits its expression. This event affects the expression of DNA methyltransferase 3A (DNMT3A), a downstream target gene of miR-29b, and induces HSC activation evidenced by increased levels of α-SMA, Collα1 and autophagy-related factors (Xie Z. et al., 2021). Moreover, SNHG7 knockdown increased the levels of miR-122-5p and reduced the mRNA and protein levels of the ribosomal protein L4 (RPL4) diminishing cell proliferation, migration, and invasion in HCC (Yang et al., 2019).

**TABLE 3:** miRNAs and lncRNAs involved in the regulation of HSCs activation and the maintenance of its fibrotic characteristics.

Cellular function	miRNA/lncRNA	Target	Signaling pathway	References
Promote HSCs activation	miR-31, miR-17-5p, miR-19, miR-27b, miR-503, miR-103-3p, miR-130a/b, and miR-942	*FIH1*, *SMAD7*, *TGFBR2*, *KLF4*, *PPAR-_γ_*	TGF-β, PI3K/AKT PPAR-_γ_	Chen et al., 2020; Hu et al., 2015; Lu et al., 2015; Tao et al., 2020; Xie X. et al., 2021; Yu et al., 2015; Zhang et al., 2016; Zhu et al., 2018
	LncRNA-ATB, HOTTIP, SNHG7, PVT1	*TGFBR1/2*, *SMAD2/3* *DNMT3A*, *PTCH1*	TGF-β, Wnt/β-catenin	Fu et al., 2016; Han L. et al., 2020; Li Z. et al., 2018
Inhibit HSCs activation	miR-98, miR-30, miR-130a-3p, miR-146a-5p, miR-200a, miR-489-3p, miR-708, sja-miR-71a	*HLF*, *KLF11*, *TGFBR1/2*, *WNT1*, *WNT5*α, *GLI3*, *JAG1*, *ZEB1*, *SEMA4D*	TGF-β PI3K/AKT Wnt/β-catenin PPAR-_γ_, JAG1/Notch3 Hedgehog	Du et al., 2015; Li L. et al., 2020; Li et al., 2021; Liu et al., 2021; Tu et al., 2015; Wang L. et al., 2020; Wang Q. et al., 2020; Yang et al., 2020
	LincRNA-p21	p21	Wnt/β-catenin	Yu et al., 2017
Promote fibrotic features in HSCs (increase expression of α-SMA, Col1A1, and accumulation of extracellular matrix)	sja-miR-1, mir-140-3p, miR-195-3p	*SFRP1*, *PTEN*	Wnt/β-catenin PI3K/AKT	Wang L. et al., 2020; Wang A. et al., 2022; Wu et al., 2019
	LncRNA-ATB, SNHG7, PVT1	*TGBR2*, *DNMT3A*, *PTCH1*	TGF-β	Fu et al., 2016

It has been reported that downregulation of PVT1 inhibits HSC activation and proliferation *in vitro* and attenuates collagen deposits *in vivo* by rescuing demethylation and overexpression of Patched1 (PTCH1) caused by miR-152 (Zheng et al., 2016).

Human cirrhotic liver and murine models of cirrhosis show a marked reduction in the long intergenic non-coding RNA-p21 (lincRNA-p21) (Jiang et al., 2024). Particularly, lentivirus-mediated lincRNA-p21 transfer into mice decreased the severity of liver fibrosis *in vivo*; the enhancement of p21 mRNA and protein expression inhibits proliferation, and reverses the activation of HSCs to their quiescent phenotype, reducing α-SMA and Col1A1 expression (Zheng et al., 2015). The suggested mechanism is that lincRNA-p21 suppresses HSC activation via suppression of the miR-17-5p-mediated-Wnt/β-catenin pathway (Yu et al., 2017; Table 3).

### 5.2 microRNAs and lncRNAs implicated in astrocytic activation

Astrocytic activation is also regulated by post-transcriptional modulators. The upregulated levels of miRNA-125b have been reported in human astrocytes in an *in vitro* model of astrogliosis induced by interleukin-6 treatment. High levels of miRNA-125b are positively correlated with the glial cell markers, GFAP, and meanwhile exogenous treatment with anti-miRNA-125b attenuates glial proliferation by increasing the expression of the cyclin-dependent kinase inhibitor 2A (CDKN2A) (Pogue et al., 2010; Table 4). The p16*^INK4A^* protein, encoded by the CDKN2A, is inactivated by promoter methylation in astrocytomas and gliomas (Alves et al., 2013; Fueyo et al., 1996), which is related to the age and sex of patients, showing a predominance of methylated CDKN2A in astrocytic tumor tissue of young female patients (Alves et al., 2013). Another study in normal human astrocytes but stimulated with lipopolysaccharide (LPS) reported that miR-211 inhibited the brain-derived neurotrophic factor (BDNF) expression by binding to the 3′-UTR of BDNF. miR-211 significantly downregulates BDNF mRNA and protein expression, thereby suppressing reactive astrocytic proliferation via the PI3K/AKT pathway (Zhang et al., 2017). Similarly, BDNF is a direct target of miR-140, which binds to the 3′-UTR of BDNF and attenuates the effects of LPS-induced injury in human astroglial cultures. Furthermore, ectopic miR-140 expression may lead to a restoration of the expression of IL-6 and TGF-α (Tu et al., 2017; Table 4). A negative regulation in astrogliosis has been reported following the induced overexpression of miR-145, achieved through a lentivirus-mediated pre-miRNA delivery system utilizing the promoter of GFAP. Astrocyte-specific overexpression of miR-145 also attenuates the morphological changes of reactive astrocytes, as well as cell proliferation and migration (Table 4). Since overexpression of miR-145 suppresses the maturation of astrocytes derived from glial progenitors, GFAP and c-myc have been suggested as potential targets of miR-145 to reduce hypertrophic reactivity through the p38 MAPK and ERK1/2 signaling pathways (Wang et al., 2015). The upregulation of GFAP, hypertrophy of the cell body, astrogliosis, and deficits in dendritic spine formation have been reported in the lateral septal nucleus and cortex in Dicer-null transgenic mice. The molecular mechanism involves the downregulation of miRNA-324-5p, followed by elevated astrocytic secretion of chemokine ligand 5 (CCL5) and downstream inhibition of the MAPK/CREB signaling pathway, leading to dysfunction in astrocyte-neuron crosstalk in a long-lasting manner (Sun et al., 2019; Table 4).

**TABLE 4:** miRNAs and lncRNAs involved in the regulation of astrogliosis.

Cellular function	miRNA/lncRNA	Target	Signaling pathway	References
Promote astrogliosis	miRNA-125b, miRNA-211, miRNA-324-5p	*CDKN2A*, *BDNF*, *CCL5*	CDKN2A/p16^INK4A^, PI3K/Akt MAPK/CREB	Alves et al., 2013; Sun et al., 2019; Zhang et al., 2017
	H19	*STAT3*, *C-MYC*	JAK/STAT	Han et al., 2018
Inhibit astrocytic activation	miR-140, miR-145, miR-1-3p	*BDNF*, *TGF*-α, *GFAP*, *C-MYC*, *CCL2*, *TNF*-α	PI3K/AKT p38 MAPK, ERK1/2	Li P. et al., 2020; Tu et al., 2017; Wang et al., 2015
	PRDM16-DT/ Prdm16os, UCA1	GLAST, MCT4	Rest/PRC2 JAK/STAT	Schröder et al., 2024; Wang H. et al., 2020

It has been reported that lncRNAs play a role in the regulation of inflammatory responses, microglial apoptosis, microglial pyroptosis, microglial activation, neuronal damage, and neuronal apoptosis in neurocognitive diseases (Chen M. et al., 2021). In this section, we describe the regulatory mechanisms of lncRNAs related to astrocyte dysfunction, mainly reactive astrogliosis.

The overexpression of the lncRNA H19 has been observed in glioblastoma tissue, and it is associated with glioma angiogenesis and invasion of glioma cells (Jia et al., 2016; Jiang et al., 2016). Overexpression of H19 through an adeno-associated viral vector delivery system in a rat epilepsy model induced the activation of hippocampal astrocytes and the release of proinflammatory cytokines, including IL-1ß, IL-6, and TNF-α by promoting the expression of Stat3 and c-Myc via JAK/STAT signaling (Han et al., 2018). Moreover, H19 may act as ceRNA and competitively bind to miRNA let-7 and suppress its expression (Kallen et al., 2013) promoting changes in the morphology and proliferation of hippocampal astrocytes and epileptic seizures by targeting Stat3. This suggests the involvement of JAK/STAT signaling pathway during the activation of astrocytes in epileptogenesis (Han C. L. et al., 2020; Table 4). Importantly, the overexpression of miR-1-3p attenuates proliferation and activation of normal human astrocytes in an *in vitro* model of spinal cord injury induced by LPS treatment; miR-1-3p directly binds to H19 and CCL2 3’UTR, reducing the levels of IL-6, and TNF-α (Li P. et al., 2020).

The lncRNA PRDM16-DT has emerged as a key regulator of astrocytes homeostasis. PRDM16-DT in human and Prdm16os in murine models are downregulated in AD. The knockdown of PRDM16-DT in human iPSC-derived astrocytes leads to functional deficits in astrocytes and induces astrogliosis downregulating central molecules of the glutamatergic neurotransmission, such as the glutamate transporter (GLAST) and lactate transporter (MCT4) correlated with disruption in glutamate uptake and lactate release. Prdm16os and PRDM16-DT exert their effects functioning as a decoy for RE1-Silencing Transcription factor (Rest) in conjunction with the methyltransferase Polycomb Repressive Complex 2 (PRC2) (Schröder et al., 2024). Induced overexpression of the lncRNA urothelial cancer-associated 1 (UCA1) shows a protective effect on neuronal injury induced by kainic acid in rats; UCA1 inhibited KA-induced abnormal elevation of GLAST, astrocyte activation via JAK/STAT signaling pathway, moreover, cognitive deficits in epilepsy rats (Wang H. et al., 2020; Table 4).

## 6 Brain-liver axis clinical implications and therapeutic interventions

Astrocytes and hepatic stellate cells play significant roles in scar formation and the progression of cell damage in the CNS and hepatic tissue, respectively. Moreover, it has been demonstrated that HSCs regulate the blood-tissue barrier in response to liver injury through their activation (Buniatian et al., 2001). Similarly, astrocytes are essential for maintaining BBB integrity. The activation of both cell types induces inflammation and disrupts cytoskeletal architecture, followed by an increase in vascular wall permeability and the release of pro-inflammatory molecules and toxins into the bloodstream. In this context, bidirectional communication may occur between the brain and liver, contributing to the development of pathological states. By providing a comprehensive understanding of the molecules involved in the activation of both cell types, we can identify potential therapeutic targets for tissue repair to ameliorate neurocognitive and hepatic diseases.

### 6.1 GFAP

Although the expression of GFAP in human-activated HSCs remains debated, various studies suggest GFAP as a hallmark in the differentiation of HSCs into myofibroblast and in astrogliosis (Schachtrup et al., 2011). Since GFAP is the main protein in the intermediate filaments of astrocytes, it has been recognized as a prototypical marker of reactive astrocytes. However, in rat hepatic tissue, GFAP displays different expression patterns depending on the time course of liver injury; for example, GFAP is highly expressed during acute injury but decreases in chronic responses. Particularly, GFAP expression has been correlated with the fraction volume of fibrosis at early stages in human cirrhotic tissue, when GFAP-positive HSCs are still negative to α-SMA, a marker of activated HSCs (Carotti et al., 2008). In this context, no relationship has been observed between α-SMA expression and fibrosis stage in patients with chronic hepatitis infection, but a correlation between GFAP and the fibrotic score (Levy et al., 2002). Moreover, GFAP immunoreactivity was positively correlated with fibrosis progression in post-transplant recurrent hepatitis C (Carotti et al., 2008). These clinical studies suggest GFAP as an early marker of activated HSCs. However, further studies are necessary to understand (1) whether GFAP-positive cells are precursors of activated HSCs (α-SMA-positive), (2) if similar GFAP expression patterns occur in liver injury with different etiologies, and (3) whether GFAP-positive cells are confined to specific areas within the liver across different stages of disease.

### 6.2 miR-455-3p

The small non-coding miR-455-3p has been described as a potential biomarker and therapeutic candidate for AD and liver fibrosis (Islam et al., 2024; Wei et al., 2019). MiR-455-3p is one of the two isoforms of mirR-455; its precursor sequence is transcribed from intron 10 of the human Col27a1 gene (collagen type XXVII alpha chain). However, the regulation of AD-related genes and hepatic fibrosis-related genes by miR-455-3p genes display opposite regulation patterns. Upregulation of miR455-3p has been observed in serum and postmortem cerebral cortex and hippocampus from AD patients. Although its molecular mechanism in reactive astrocytes is still not well understood, it has been reported that miR-455-3p knockout mice exhibit increased activity in astrocytes and microglia (Islam et al., 2024).

During HSCs activation, miR455-3p is significantly downregulated. Its reduction has been linked to liver fibrosis in various mouse models of liver injury such as bile duct ligation, high-fat diet, and CCl_4_ administration (Wei et al., 2019). Ectopic overexpression of miR455-3p inhibits HSC activation by suppressing heat shock factor 1 (HSF1), which is involved in the Hsp47/TGF-β/Smad4 signaling pathway in liver tissue (Wei et al., 2019). Although miR455-3p has potential as a therapeutic target, further studies are needed to elucidate its specific role in astrocytes/HSCs activation across different etiologies, in order to determine its potential as a biomarker for scar formation and neuronal and hepatic regeneration.

### 6.3 miR-140

The axis miR-140/BDNF has been suggested as a promising target to ameliorate reactive human astrocyte proliferation after spinal cord injury (Tu et al., 2017). MiR-140 binds to the 3′UTR of BDNF and inhibits its expression. Since BDNF upregulation regulates astrocyte proliferation and differentiation, ectopic expression of miR-140 restores BDNF and pro-inflammatory cytokine levels, thereby ameliorating astrogliosis after nerve fiber damage (Tu et al., 2017). The miR140-3p belongs to the miR-140 cluster and has been linked to liver fibrosis. Upregulation of miR-140-3p is correlated with activation of rat HSCs (HSC-T6) through silencing of the tumor suppressor, phosphatase and tensin homolog deleted on chromosome 10 (PTEN), which enhances activated HSC proliferation and reduces apoptosis via AKT/mTOR signaling pathway. Meanwhile, miR-140-3p knockdown results in downregulation of α-SMA and desmin levels (Wu et al., 2019), demonstrating the potential of miR140-3p to suppress the fibrotic role of TGF-β1.

Clinical data showed that miRNA-140 is significantly downregulated in liver tissues of patients with HCC and might stimulate metastasis and HCC progression (Kong et al., 2021), analog to reported in HCC mouse models (Ghafouri-Fard et al., 2021). The molecular mechanism has been described by *in vitro* studies that demonstrated miRNA-140 overexpression inhibits cell proliferation, migration and invasion in HCC through PI3K Akt signaling pathway, TGF-β signaling pathway, and MAPK signaling pathway (Kong et al., 2021; Lu et al., 2020). Importantly, studies in tumor xenograft mice model indicated that the downregulation of miRNA-140 leads to sorafenib resistant and poor prognosis in HCC, this chemoresistance might be regulated through the small nucleolar RNA host gene 16, this lncRNA is overexpressed and its directly interacting with miRNA-14, which targets the pregnane X receptor resulted in the modulation of downstream genes involved in drug-resistant during HCC (Li J. et al., 2018; Ye et al., 2019).

The prognostic role of miRNA-140 has been identified in cancerous brain tumor as well. Downregulation of miRNA-140 has been inversely associated with the cysteine protease, cathepsin B expression in glioblastoma multiforme (Ho et al., 2019). In this case, miRNA-140 might regulate mesenchymal transition and response to temozolomide, the first-line antineoplastic against glioblastoma multiforme via cathepsin B (Ho et al., 2019; Palizkaran Yazdi et al., 2024). Additionally, treatment with cathepsin B inhibitors such as E64D and CA074Me reduces glioma cell proliferation, reduces amyloid plaques deposition, protects against astrocytic apoptosis, rescues motor and cognitive dysfunction in animal models (Cawley et al., 2014; Xu et al., 2014). Cathepsin B is an important mediator of NLR family protein domain containing 3 inflammasome activation, which have implications in a variety of neurodegenerative diseases such as Parkinson and AD but in the progression of liver diseases by inflammatory response-mediated HSC activation and fibrogenesis (Charan et al., 2023).

### 6.4 miR-148a-3p

The miR-148a-3p has been associated with neuroprotection by the inhibition of proinflammatory factors (Ramírez et al., 2022). Qian et al. (2024) demonstrated that miR-148a-3p from astrocytes-derived exosomes stimulates phenotype transition of microglia *in vitro* and in traumatic brain injury model in rats. The transfection of miR-148a-3p induces the polarization into the pro-inflammatory M1 phenotype to the anti-inflammatory M2 phenotype in pre-microglia cultures. Additionally, miR-148a-3p attenuates lipopolysaccharide-mediated inflammatory response *in vitro* and improves the modified neurological severity score after traumatic brain injury in rat model, this neurological restoration occurs via the inhibition of the nuclear factor kß pathway (Qian et al., 2024).

Similarly, the role of miR-148a-3p in HCC progression has been demonstrated in xenograft liver cancer model (Zhang et al., 2022). miR-148a-3p is downregulated in the transformation process from qHSCs to activate HSCs, *in vitro* co-cultures showed that decreased exosomal miR-148a-3p may be uptake by HCC cells leading to tumorigenesis and HCC progression, this data is correlated with the result from clinical samples that show the downregulation of miR-148a-3p in patients with primary HCC tumor (Cheng et al., 2017; Gailhouste et al., 2013; Zhang et al., 2022). In healthy microenvironment, miR-148a-3p displays high expression in hepatic satellite cells and HSCs, thus miR-148a-3p overloaded exosomes might be a promising approach to regulate the proliferation and invasion of tumor microenvironment via ITGA5/PI3K/Akt pathway and improve prognosis (Zhang et al., 2022).

### 6.5 lncRNA HOTTIP

As previously discussed in Section “4 The atlas of astrocytes in nervous system diseases,” the LncRNA HOTTIP mediates HSCs activation. Interestingly, aberrant HOTTIP down-regulation has been demonstrated in various glioma cell lines isolated from human brain tissue such as A172, U251, U-118 MG and U-87 MG (Xu et al., 2016). Although the specific role of HOTTIP in astrocytes has not been described, its role by regulating microglia-mediated inflammation and neuronal damage has been reported (Lun et al., 2022). The overexpression of HOTTIP in an intro model of PD using 1-Methyl-4-phenylpyridium showed microglial activation, exacerbating proinflammatory cytokine expression such as IL-lβ, IL-6, IL-18, TNF-α, iNOS, COX2, and phosphorylated NF-κB. Similarly, an induced PD mouse model combined with HOTTIP knockdown confirmed the suppression of MPTP-induced NLRP3-ASC-Caspase-1 inflammasome activation and microglial activation in substantia nigra, additionally, HOTTIP knockdown rescues the dopamine content in striatal brain region and improvement in motor and cognitive function (Lun et al., 2022). Future studies are needed to address the possible role of HOTTIP in astroglia and its clinical significance to ameliorate another neurocognitive disease.

## 7 Conclusion and future perspectives

The development of novel therapeutic agents for neuronal and hepatic diseases remains a challenge. The transcriptome, including both coding and non-coding transcripts in HCSs and astrocytes, provides a deeper understanding of the molecules involved in the activation of both cell types and their contribution to pathological stages. Preclinical trials are necessary to address challenges in the delivery system of agonists or antagonists (i.e., ectopic miRNA) to the target tissue, ensuring the stability of the molecules while minimizing innate immune response.

Exosomes have been suggested as novel drug delivery to target cells (Qian et al., 2024; Zhang et al., 2022), these nano extracellular vesicles secreted by qHSCs and healthy astrocytes containing specific bioactive molecules might recover the physiological neuro and liver microenvironment acting as anti-inflammatory and tumor-suppressor molecules. The use of three-dimensional organ-like architecture, called “organoid culture” is an innovative approach to face the challenge of study the molecular mechanism in the crosstalk between the brain-liver axis (Liu et al., 2024) and validate the role of circulating biomolecules (i.e., exosomes) in both systems. For example, differentiation of induced pluripotent and embryonic stem cells to develop brain organoids expressing both excitatory and inhibitory neurons and all the glial cell types, microglia, oligodendrocytes and astrocytes represent a potential tool to understand the glial microenvironment not only in the human brain development but in neurological disease (Agarwal et al., 2021; Heydari et al., 2021). Organoid model generated from co-cultures of human induced pluripotent stem cells-derived hepatocytes, mesenchymal stem cells from mouse or human, and human umbilical vein endothelial cells have been developed to study various liver diseases such as non-alcoholic liver disease, fibrosis or chronic viral hepatitis (Heydari et al., 2021). However, the development of brain-liver axis models results in a complex system that might consider the neuronal, endocrine and immunological communication between both systems. For example, an engineered model of gut-liver-brain axis has been used to study the interaction between these systems in PD (Trapecar et al., 2021). The microphysiological systems are linked sharing a common culture media containing circulating cells in a continues coculture to emulate a colon mucosal barrier incorporating innate immune cells where the microbiome adsorption and metabolize occurs resulting in the production and release of signaling biomolecules, which are transported to the liver through the portal circulation to reach the hepatocytes, the microbiome can influence the Kupffer cells in the liver, all the inflammatory mediators soluble biomolecules and metabolites are transport to the brain through systemic circulation to finally reach the migration of adaptative immune CD4^++^ T cells via systemic circulation between the three systems (Trapecar et al., 2021). An organoid model of the brain-liver axis might help to face the limitations of the current *in vitro* and animal models to study the communication between both systems. For instance, the liver innervation displays differences across species, nerves fibers are in contact directly with hepatocytes in human, monkey and rabbit but not in rat (Miller et al., 2021). Moreover, it has been reported brain-region differences in the electrophysiological properties of astrocytes, their calcium dynamics and gap junction coupling; this physiological heterogeneity must be considered in future models (Zhang and Barres, 2010). However, taken together, the intercommunication within the brain-liver axis suggests that combination therapy could be a promising approach for tissue repair in neuronal and hepatic conditions.
